# Clearing the Fog: Is Hydroxychloroquine Effective in Reducing Coronavirus Disease-2019 Progression? A Randomized Controlled Trial

**DOI:** 10.7759/cureus.14186

**Published:** 2021-03-30

**Authors:** Sultan M Kamran, Hussain Abdul Moeed, Zill-e-Humayun Mirza, Arshad Naseem, Rizwan Azam, Naqeeb Ullah, Farrukh Saeed, Wasim Alamgir, Salman Saleem, Shazia Nisar

**Affiliations:** 1 Pulmonology and Critical Care, Pak Emirates Military Hospital, Rawalpindi, PAK; 2 Pulmonology, Pak Emirates Military Hospital, Rawalpindi, PAK; 3 Gastroenterology, Pak Emirates Military Hospital, Rawalpindi, PAK; 4 Critical Care, Pak Emirates Military Hospital, Rawalpindi, PAK; 5 Infectious Disease, Pak Emirates Military Hospital, Rawalpindi, PAK; 6 Internal Medicine, Army Medical College, Rawalpindi, PAK; 7 Internal Medicine, Pak Emirates Military Hospital, Rawalpindi, PAK

**Keywords:** covid-19, viral clearance, disease progression, hcq, rct, mild infection

## Abstract

Background

Hydroxychloroquine (HCQ) has been considered for the treatment of coronavirus disease 2019 (COVID-19), but data on its efficacy are conflicting. We analyzed the efficacy of HCQ along with standard of care (SOC) treatment, compared with SOC alone, in reducing disease progression in mild COVID-19.

Methods

A single-center open-label randomized controlled trial was conducted from April 10 to May 31, 2020 at Pak Emirates Military Hospital, Rawalpindi. Five hundred patients of both genders between the ages of 18 and 80 years with mild COVID-19 were enrolled in the study. A total of 349 patients were assigned to the intervention group (standard dose of HCQ plus SOC) and 151 patients were assigned to SOC only. The primary outcome was progression of disease while secondary outcome was polymerase chain reaction (PCR) negativity on days 7 and 14. The results were analyzed on Statistical Package for Social Sciences (SPSS; IBM Corp., Armonk, NY) version 23. A p-value <0.05 was considered significant.

Results

The median age of the intervention group was 34 ± 11.778 years and control group was 34 ± 9.813 years. Disease progressed in 16 patients, 11 (3.15%) of which were in the intervention group and 5 (3.3%) in the control group (p-value = 0.940). PCR negative cases in intervention and control groups on day 7 were 182 (52.1%) and 54 (35.8%), respectively (p-value = 0.001); and on day 14 were 244 (69.9%) and 110 (72.9%), respectively (p-value = 0.508). Consecutive PCR negativity on days 7 and 14 was observed in 240 (68.8%) patients in the intervention group compared to 106 (70.2%) in the control group (p-value = 0.321).

Conclusion

The addition of HCQ to SOC in hospitalized mild COVID-19 patients neither stops disease progression nor helps in early and sustained viral clearance.

## Introduction

Beyond supportive care, there are currently no proven treatment options for coronavirus disease 2019 (COVID-19) [1]. As mortality in patients with critical disease category is substantial [2], every effort has to be made to intervene early and aggressively to prevent the progression of disease. Globally, approximately 113 million confirmed cases of COVID-19 have been reported with a case fatality ratio of 2.2% [3]. Nevertheless, data from various international studies show that 81% of patients have had mild to moderate disease, which includes non-pneumonia and pneumonia cases [4]. Since asymptomatic carriers and patients with mild disease are the main sources of disease transmissibility [5], the management of mild disease is equally important. Therefore, it is a matter of utmost importance to detect mild cases earlier and start some investigational treatment in carefully selected hospitalized patients. 

According to the severity of the disease process, various experimental treatment options have been applied. Out of many therapeutic off-label options, hydroxychloroquine (HCQ) seems more suitable owing to its known safety profile, side effects, posology, and drug interactions [6]. HCQ has had good in vitro activity against severe acute respiratory syndrome coronavirus 2 (SARS-CoV-2) and better safety profile than chloroquine [7,8]. A small study on 36 patients showed that HCQ treatment is significantly associated with viral load reduction and disappearance in patients with COVID-19 [9]. Additionally, it has been hypothesized that HCQ inhibits cytokine release storm by reducing CD-154 expression in T cells, thus reducing chances of disease progression [10]. 

The therapeutic role of HCQ can be studied in various aspects of COVID-19 infection. This includes the time for virologic clearance, worsening of symptoms, and biochemical markets of cytokine release storm. In Pakistan, Pak Emirates Military Hospital (PEMH) is the largest COVID-19 designated hospital in the country. This hospital has treated more than 3000 patients with COVID-19, including many asymptomatic and mild cases. On the basis of limited evidence available, HCQ was administered to hospitalized patients with mild COVID-19 after obtaining informed consent with the objective of achieving early viral clearance and preventing progression of disease.

This article was previously posted to the medRxiv preprint server on October 11, 2020 (https://www.medrxiv.org/content/10.1101/2020.07.30.20165365v2.full?).

## Materials and methods

This single-center, parallel, open-label randomized controlled trial (RCT) was conducted from April 10 to May 31, 2020 in the department of pulmonology, PEMH and included more than 500 patients between the ages of 18 and 80 years. The study design was approved by institutional Ethical Review Committee. The study population consisted of patients from both genders with mild confirmed COVID-19 after their approved written consent. The study protocol and approval documents are available online at ClinicalTrials.gov with trial number of NCT04491994.

Sample size was calculated using Open Source Epidemiologic Statistics for Public Health (OpenEpi.com) with a 95% confidence level, 80% power to detect a difference, and an enrollment ratio of 2:1 between the intervention and control groups. A two-sided significance level of α =0.05 was set for seven days in the median time to clinical improvement between the two groups, assuming that the median time in the standard of care (SOC) treatment group was 14 days, and assuming 55% efficacy of HCQ in preventing disease progression and achieving viral clearance on day 7. The calculated sample size was 467.

**Figure 1:**
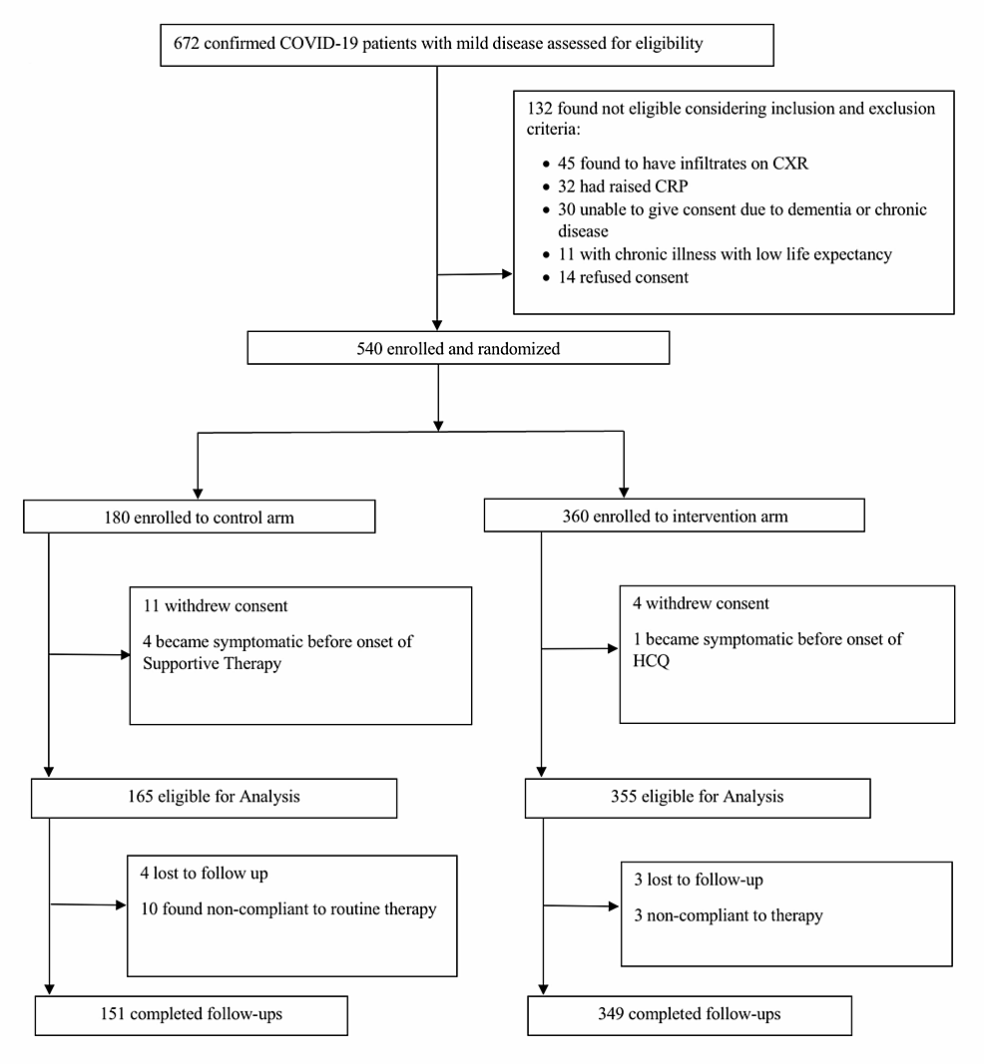
Randomization flow sheet COVID-19: coronavirus disease 2019; CXR: chest X-ray; CRP: C-reactive protein; HCQ: hydroxychloroquine

During the study period, 672 confirmed reverse transcriptase-polymerase chain reaction (RT-PCR) positive cases were assessed for eligibility. Inclusion criteria included (I) mild COVID-19, (II) RT-PCR-confirmed infection, (III) hospital-admitted patients, and (IV) 18-80 years of age. Exclusion criteria were (I) moderate, severe, or critical COVID-19; (II) day-zero C-reactive protein (CRP) >6 mg/dl or absolute lymphocyte count (ALC) <1000; (III) evidence of infiltrates on X-ray chest; (IV) co-morbidity with life expectancy of less than six months; or (V) contraindications to HCQ therapy. The severity of disease was defined in accordance with the criteria devised by the World Health Organization [11]. Mild disease was defined as patients with uncomplicated upper respiratory tract viral infection having nonspecific symptoms such as low-grade fever (fever <100°F for <3 days), fatigue, body aches, cough (with or without sputum production), anorexia, muscle pain, sore throat, nasal congestion, anosmia, headache, and rarely diarrhea, nausea, and vomiting. Any chronic health condition for which patients were on prior treatment was considered a co-morbidity. One hundred and thirty-two cases did not meet the selection criteria and were subsequently excluded. Five hundred and forty patients were then enrolled and randomized. Further 20 patients were excluded from analysis as 15 withdrew consent and five became symptomatic before the first dose of HCQ. During the follow-up, 13 patients deviated from the advised therapy and seven were lost to follow-up, yielding a final study population of 500 (Figure 1).

Randomization rules were designed by Dr Wasim Alamgir together with principal investigators and were implemented by an independent statistician who was not involved in data analysis. Stratified random sampling was used to stratify all eligible patients according to age, gender, and co-morbidities. A computerized random number generator was used and the allocation was done in 2:1 sequence. Cards with each group assignment number randomly generated by the computer were placed in sequentially numbered envelopes, which were opened as the patients were enrolled.

A total of 349 patients were enrolled in the intervention group and were given HCQ in addition to SOC. The standard dose of HCQ was 400 mg by mouth twice a day for day 1 followed by 200 mg 12 hourly for the next five days. The patients who did not give consent for treatment with HCQ or had a known allergy to HCQ or chloroquine or had any other known contraindication to treatment with the study drug (retinopathy, glucose 6-phosphate dehydrogenase deficiency, and QT prolongation) served as controls. Controls were matched with participants on the basis of age, gender, and co-morbidities. They comprised 151 patients and were given SOC alone. SOC treatment comprised daily oral vitamin C (2 g), oral zinc (50 mg), oral vitamin D (alfacalcidol 1 µg), and oral acetaminophen (for body aches and fever).

Data regarding age, co-morbidities, history of contact with a positive patient, days since contact, duration of symptoms, COVID-19 RT-PCR status with date, baseline laboratory tests, and X-ray chest were recorded for all patients. Six-hourly body temperature, respiratory rate, resting oxygen saturation with pulse oximetry, 10 feet oxygen desaturation, ALC, and CRP were monitored daily during hospitalization. Chest X-rays on days 0, 3, and 5 were also done to check for any infiltrates. Patients on HCQ underwent daily electrocardiography to assess QT prolongation, where an increase in QT interval >25% from baseline was considered significant to withdraw HCQ. Any visual complaint by the patient warranted urgent referral to ophthalmologist to consider the stoppage of the drug. Other side effects of the study drug, such as headache, nausea, vomiting, abdominal pain, skin reactions, vertigo, deafness, psychosis, and anemia, were also monitored. No patient, investigator, and statistician was masked to treatment assignment. Laboratory workers who performed sampling for COVID-19 RT-PCR, basic blood tests, and other routine measurements were unaware of treatment information.

The primary outcome was disease progression within five days of start of treatment. Progression of disease was defined by the development of fever >101°F for >72 hours, shortness of breath with minimal exertion, derangement of basic laboratory parameters (ALC < 1000 or raised CRP), or appearance of infiltrates on X-ray chest. The secondary outcome was COVID-19 viral clearance for which RT-PCR status was recorded on days 7 and 14 after the initiation of treatment.

Statistical interpretation of data was performed using Statistical Package for Social Sciences (SPSS) version 23 (IBM Corp., Armonk, NY). Results were expressed as mean and standard deviation (±SD) for all continuous variables, and as frequency and percentage for categorical data. Significance was assessed using t-test and chi-squared tests as appropriate to the nature and distribution of the variables. A p-value <0.05 was considered statistically significant.

## Results

During the study, 500 patients with mild COVID-19 were included, with a mean age of 35.96 ± 11.2 years (intervention group: 34 ± 11.778 versus control group: 34 ± 9.813). Overall, 466 (93.2%) males and 34 (6.8%) females were included in the trial. Male-to-female proportion in the intervention and control groups was 328 (94%) males and 21 (6%) females versus 139 (91.4%) males and 13 (8.6%) females, respectively. Most patients were healthy young individuals with co-morbidities only in 38 (7.6%), where 31 (8.9%) belonged to the intervention group and seven (4.6%) to the control group. Type 2 diabetes mellitus was the most common co-morbidity (3%). Positive contact history was found in 315 (63%) patients. Among constitutional symptoms, cough (32.6%), low-grade fever (26.6%), body aches (19.2%), anosmia (16.6%), and fatigue (11.2%) were the most common. Less common symptoms were sore throat (6.6%), diarrhea (4.2%), and headache (4.2%). Completely asymptomatic patients were 101 (20.2%). HCQ in addition to SOC treatment was given to the intervention group comprising 349 (69.8%) patients, while 151 (30.2%) patients in the control group received only SOC treatment.

Among the 16 patients who showed disease progression (Table 1), 11 (3.15%) were from the intervention group, and five (3.3%) from the control group (p-value = 0.940). In the intervention group, out of 11 patients with disease progression, 4/31 (12.9%) had co-morbidities, as compared to 2/7 (28.6%) in the control group (p-value = 0.304). Progression of disease was significantly associated with the presence of co-morbidities, as six (15.8%) patients out of 38 with co-morbidities showed progression, in contrast to only 10 (2.2%) out of 462 patients without co-morbidities (p-value < 0.00001).

**Table 1:** Assessment of effect of HCQ on progression of disease HCQ: hydroxychloroquine; SOC: standard of care

Effect of HCQ	Treatment		p-Value
	HCQ plus SOC	SOC alone	
Overall progression	11/349 (3.15%)	5/151 (3.3%)	0.940
Progression in patients with co-morbidities	4/31 (12.9%)	2/7 (28.6%)	0.304

COVID-19 RT-PCR negativity was observed in 236 (47.2%) patients on day 7 and in 354 (70.8%) patients on day 14. Effect of HCQ on RT-PCR status of the study population is given in Table 2. Daywise RT-PCR negativity in the intervention and control groups, respectively, was as follows: day 7: 182 (52.1%) versus 54 (35.8%) (p-value = 0.001), day 14: 244 (69.9%) versus 110 (72.9%) (p-value = 0.508). Successive seventh and 14th day RT-PCR negativity was observed in 240 (68.8%) patients in the intervention group versus 106 (70.2%) patients in the control group (p-value = 0.321). RT-PCR status remained positive in 62 (17.8%) patients of the intervention group versus 32 (21.2%) patients of the control group (p-value = 0.321).

**Table 2:** Assessment of effect of HCQ on RT-PCR status of study population HCQ: hydroxychloroquine; RT-PCR: reverse transcriptase-polymerase chain reaction

	Treatment		p-Value
	Intervention group n = 349	Control group n = 151	
RT-PCR on day 7			
Negative	182 (52.1%)	54 (35.8%)	0.001
Positive	167 (47.9%)	97 (64.2%)	
RT-PCR on day 14			
Negative	244 (69.9%)	110 (72.9%)	0.508
Positive	105 (30.1%)	41 (27.2%)	
RT-PCR negativity on days 7 and 14	240 (68.8%)	106 (70.2%)	0.321
RT- PCR positivity on days 7 and 14	62 (17.8%)	32 (21.2%)	
RT-PCR negative on day 7 but positive on day 14	36 (10.3%)	8 (5.3%)	

## Discussion

Although there was a significant promotion of the effectiveness of HCQ in treating COVID-19, our study did not show any notable benefit of using HCQ. First, HCQ did not prevent the progression of disease in patients with or without co-morbidities, though it was postulated to dampen the cytokine release storm by Zhou et al. [10]. Second, its addition to supportive treatment showed significantly better early RT-PCR negativity on day 7, but on day 14, there was no noteworthy difference in RT-PCR negativity between the two study groups. Nonetheless, HCQ did not show any side effects in our study. We used the same doses of the study drug as used by Yao et al. [12] and no side effects were observed in their study as well.

Results of our study are also contrary to a highly publicized study by Gao J et al. [13], which showed early viral clearance and decreased rate of disease progression. Compared to our study’s sample size of 500 patients, Gao et al.'s study had a smaller sample size (n = 100) and they used chloroquine instead of HCQ. As far as viral clearance on day 7 is concerned, our results are similar to those of the nonrandomized controlled trial from France by Gautret et al. [9]. Their study showed significantly better viral clearance on day 6 of inclusion (70% versus 12.5%; p-value = 0.001) with use of 600 mg/day of HCQ for 10 days. However, in addition to HCQ, they also used azithromycin, and although highly rated initially, their study only had 20 participants in intervention arm, out of which, six were removed due to intolerance to medication. Additionally, it was a nonrandomized trial containing major biases between study groups, and patients were not followed until day 14 to assess viral clearance again. In contrast, we followed patients until day 14 and found that a subset of day 7 RT-PCR negatives turned positive again on day 14. This observation found in our study might be because of false-negative RT-PCR on day 7 owing to variable sensitivities of testing kits or a false-positive RT-PCR on day 14 due to the presence of noninfectious dead viral particles. When we compare results of our study with the RCT done by Chen et al. [14], interestingly it is found that although day 7 RT-PCR results of our study are showing a clear edge to HCQ, the primary endpoints in both studies are similar. Chen et al. used the same dose of HCQ as in our study, but in moderate COVID-19. Their study showed that HCQ did not prevent disease progression and there was similar viral clearance between the supportive treatment group and HCQ group (93.3% versus 86.7%; p-value > 0.05) on day 7. However, sample size only included 30 patients and RT-PCR status was not checked on day 14.

Our study also demonstrates similar results as recommended by the Infectious Diseases Society of America by Bhimraj et al. [15]. They analyzed three RCTs and six comparative cohort studies done on confirmed patients with COVID-19 who were hospitalized and treated with HCQ. They studied many variables such as mortality, clinical progression, clinical improvement, and adverse events, and concluded that HCQ failed to show any benefit in terms of viral clearance or halting progression of disease. In our study, disease progression was significantly higher in patients with co-morbidities even at younger age. This observation was also proven in a large-scale study that had demonstrated that patients with chronic diseases are at a higher risk of disease progression [16].

It has been seen that coronaviruses such as SARS coronavirus and Middle East respiratory syndrome coronavirus predominantly affect male gender [17]. We found that 93.2% of population infected with SARS-CoV-2 in our study was also male. Since, at the start of COVID-19 pandemic in Pakistan, our hospital had the policy to admit every RT-PCR positive case, the median age of our study population was relatively younger.

Nevertheless, there are certain limitations in our study as well. First, the main subgroup in which the study was conducted were males, so the results cannot be generalized to both genders. Second, the study was conducted in mild cases, and moderate/severe cases were not included, so it cannot be determined whether HCQ is of any benefit in advanced COVID-19 or not. Third, the patients were not followed up after discharge from the hospital; hence, correct progression of disease could not be ascertained. Fourth, we did not use quantitative RT-PCR to determine the viral load, which is a strong bias to affect viral clearance. Fifth, RT-PCR positivity on day 14 is of uncertain significance because it is now evident that after 10th day of the onset of illness, the presence of nonreplicable viral nucleic acid material is being picked up by the RT-PCR [18,19], and such patients are regarded as noninfectious. Finally, even with the best sampling techniques, the sensitivity of RT-PCR for SARS-CoV-2 ranges between 34% and 80% [20]; therefore, clear estimation of viral clearance remains under question. Despite these limitations, our study is the first of its own kind in Pakistan, which is reinforced by a larger sample size and a relatively longer follow-up time.

## Conclusions

The study shows that addition of HCQ to supportive treatment in mild COVID-19 cases is not considerably associated with the prevention of disease progression. Despite showing significantly early RT-PCR negativity on day 7, day 14 RT-PCR results are similar to those of non-HCQ arm. The findings of our study correlate with the results of various clinical trials done internationally.
